# Atypical presentation of carcinoid tumor with unresolved right shoulder pain: a case report

**DOI:** 10.1186/1752-1947-8-142

**Published:** 2014-05-07

**Authors:** Nay Thi Tun, Rajen Oza

**Affiliations:** 1 Department of Internal Medicine, Easton Hospital, School of Medicine, Drexel University, 250 South 21st Street, Easton, PA 18042, USA; 2 Department of Hematology/Oncology, Easton Hospital, 250 South 21st Street, Easton, PA 18042, USA

**Keywords:** Metastatic carcinoid tumor, Neuroendocrine tumor, Right shoulder pain

## Abstract

**Introduction:**

Carcinoid tumors are variants of neuroendocrine tumors that typically arise from the gastrointestinal tract and the bronchus, but they can involve any organ. Unresolved right shoulder pain manifesting as the first clinical presentation of carcinoid tumor with unknown primary origin is a rare clinical entity. To the best of our knowledge, herein we present the first case report describing metastasis to the right shoulder joint in a patient who presented with bone pain as the first clinical manifestation of metastatic carcinoid tumor of unknown primary origin. Metastasis to the right scapula as the first presentation of an underlying carcinoid tumor in the primary bronchus has been reported previously.

**Case presentation:**

A 72-year-old Caucasian woman presented with pain in her right shoulder after a fall. She delayed seeking medical attention for 4 weeks for personal reasons. Her physical examination revealed no erythema or swelling of the right shoulder. However, tenderness was noted on the right subacromial bursa and the right acromioclavicular joint. Her drop arm test was positive. An X-ray of the right upper extremity showed no fracture. She did not respond to methylprednisolone injections or physical therapy. Because of the unresolved right shoulder pain with disturbance of her daily activities, magnetic resonance imaging of the right shoulder was ordered, which revealed permeative destruction of the right scapula. Because the permeative destruction of the bone could have been an osteolytic malignant feature, positron emission tomography–computed tomography was performed, which produced a scan showing osseous metastasis to the right scapula, multiple liver metastases and a 1.7cm right-lower-lobe pulmonary nodule. Her serotonin and chromogranin A levels were significantly elevated. The patient was treated with palliative cisplatin and etoposide chemotherapy followed by locoregional treatments for metastatic carcinoid tumor. She had mild improvement in her right shoulder pain, as well as better range of motion and improved quality of life, before she died less than 2 years after her diagnosis.

**Conclusion:**

Our present case report emphasizes the protean manifestations of carcinoid tumors with the importance of early diagnosis of bone metastases from these tumors, because early diagnosis plays a major role in choosing the therapeutic regimen and prognosticating the course of the disease. The treatment goals for high-grade, poorly differentiated carcinoid tumors of unknown origin are decreasing the tumor load while controlling symptoms with chemotherapy and local modality treatments.

## Introduction

Carcinoid tumor is one of the variants of neuroendocrine tumors. The incidence of carcinoid tumors is 2 per 100,000 general population [1,2]. Carcinoid tumors are often indolent tumors due to very slow growth, although some are high-grade with malignant features, which lead to high morbidity and mortality. Traditionally, carcinoid tumors are thought to derive from three main embryonic divisions of the gut: foregut carcinoids from lungs, bronchus or stomach; mid-gut carcinoids from the small intestine, appendix or proximal large bowel; and hindgut carcinoids from the distal colon or rectum [3]. Most carcinoid tumors arise from the gastrointestinal tract (65%), followed by the bronchi (25%) [4], but they can involve any organ.

Bone metastasis from carcinoid tumors was underestimated before the advancement of magnetic resonance imaging (MRI), octreotide scanning and [^123^I]metaiodobenzylguanidine and bone scintigraphy. In one study, only 12% of metastatic carcinoid tumors were found to have symptomatic bone metastases [5]. Bone pain, bone fracture and cord compression are the few possible clinical symptoms that suggest stage IV metastatic disease in patients with neuroendocrine tumors [6]. The most common metastatic area is the axial spine within the thoracic vertebrae (40%), followed by lumbar vertebrae (34%) and cervical vertebrae (32%) [7]. Less commonly, ribs and pelvic metastases are found [7]. In the present report, we describe the case of a patient with a highly aggressive malignant carcinoid tumor with an unusual presentation and without a defined primary site.

## Case presentation

**Figure 1:**
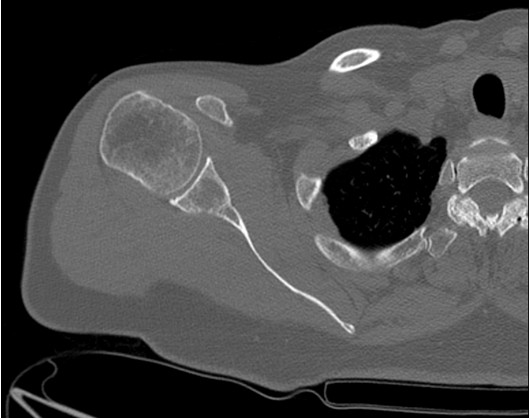
Magnetic resonance imaging scan of the patient’s right shoulder shows permeative destruction of the right scapula (black arrow).

A 72-year-old Caucasian woman presented with pain in her right shoulder after having a fall from a ladder at home. She had not sought medical attention for 4 weeks for personal reasons. At her initial presentation, she had increasing pain with limited range of motion in all directions. She had a medical history of melanoma *in situ* on her mid-back, which was status post-excision 6 years ago. She had exposure to asbestos at work of unknown duration. She had never smoked or drunk alcohol in her life. She had no weight loss, fever or night sweats. Her physical examination revealed no erythema, swelling or warmth on the right shoulder joint. However, her right subacromial bursa and acromioclavicular joint were tender. Her drop arm test was positive. She also had no abnormal skin lesions such as melanocytic nevi. An X-ray of her right upper extremity showed no fracture. She was referred to an orthopedist because of unresolved right shoulder pain. She did not respond to methylprednisolone injections. She underwent physical therapy for her right shoulder joint, but without pain relief. She also did not tolerate immobilization with a sling. MRI of her right upper extremity showed a permeative, destructive lesion of the right scapula (Figure 1).

**Figure 2:**
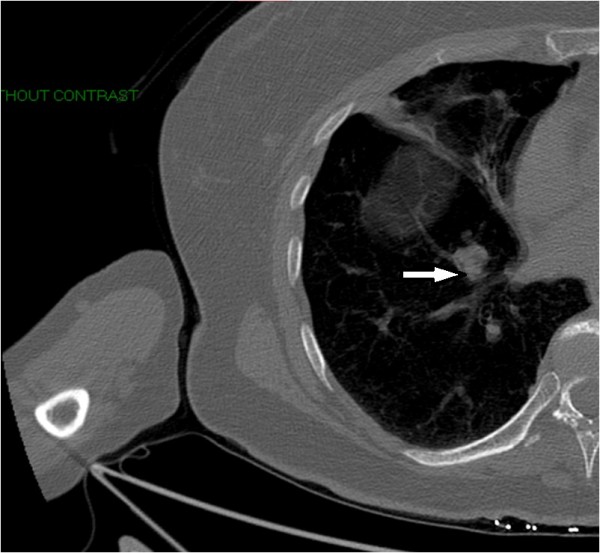
Computed tomography scan of right shoulder without intravenous contrast incidentally shows a rounded 2cm right lower lobe pulmonary mass (white arrow) with no pathologic lesion on right shoulder.

**Figure 3:**
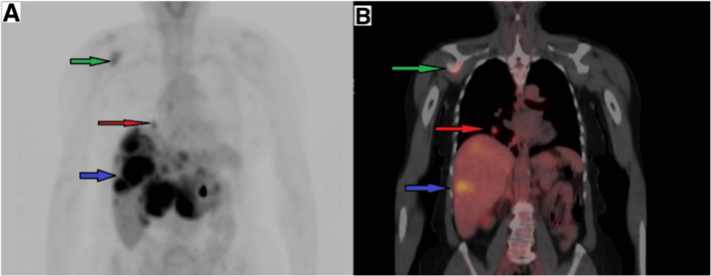
**A Position emission tomography scan in coronal view shows metastasis to right scapula (green arrow), a 1.7cm right lower lobe lung nodule (red arrow) and multiple liver metastases (blue arrow). B** Position emission tomography-computed tomography fusion scan in coronal view shows metastasis to right scapula (green arrow), a 1.7cm right lower lobe lung nodule (red arrow) and multiple liver metastases (blue arrow).

Because of persistent pain in her right shoulder, the patient underwent computed tomography (CT) of her right shoulder, which showed no fracture, dislocation or pathologic bone lesion on her right shoulder but incidentally revealed a rounded, 2cm, right lower lobe lung mass (Figure 2). As the solitary pulmonary nodule was suggestive of malignancy on chest CT, positron emission tomography-computed tomography (PET-CT) was performed, which showed osseous metastasis of the right scapula, multiple liver metastatic nodules and a 1.7cm right-lower-lobe pulmonary nodule (Figure 3A-B). The patient was then referred to an oncologist. She was found to have non-tender hepatomegaly. Therefore, referred pain to the right shoulder from underlying hepatomegaly versus bone pain from metastasis was suspected. Tumor load was found to be high in the liver and bone with a solitary malignant lung nodule. Widespread metastases of underlying cancer with an unknown primary site were found.

**Figure 4:**
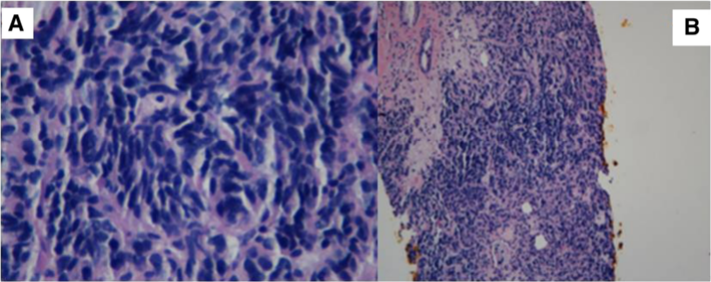
**Stained histological biopsy tissue specimens from left lobe of the liver. A**: Core biopsy tissue of the left lobe of the liver shows a poorly differentiated neuroendocrine tumor suggestive of small-cell carcinoma (original magnification, 400×). **B**: Core biopsy tissue of the left lobe of the liver shows a poorly differentiated neuroendocrine tumor suggestive of small-cell carcinoma (original magnification, 100×).

Because the liver was the most easily accessible organ with multiple metastases in this case, the patient was referred for a liver biopsy. A core biopsy of the mass in the left lobe of her liver showed a high-grade, poorly differentiated neuroendocrine carcinoma. Histologically, the differential diagnosis consisted of small-cell carcinoma (Figure 4A-B). However, immunohistochemical staining was positive for neuron-specific enolase, synaptophysin and chromogranin A in tumor cells, confirming neuroendocrine cancer.

**Figure 5:**
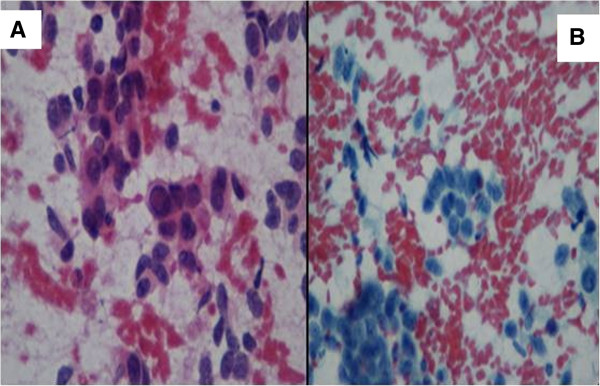
**Stained histological biopsy tissue specimens from right lower-lobe pulmonary nodule. A**: Core biopsy tissue of the right lower-lobe pulmonary nodule shows non-small-cell lung cancer features (original magnification, 400×). **B**: Core biopsy tissue of the right lower-lobe pulmonary nodule also shows small-cell carcinoma features (original magnification, 400×).

As most small-cell carcinomas may originate from the lung, the patient was recommended to undergo CT-guided biopsy of the right lung mass, which also showed poorly differentiated carcinoma with mixed small-cell and non-small-cell lung cancer features (Figure 5A-B). Immunohistochemical stains were positive for chromogranin A, synaptophysin, cytokeratin 7 and thyroid transcription factor 1 and weakly positive for cytokeratin 20.

The 24-hour urinary 5-hydroxyindoleacetic acid (5-HIAA) test result was 42mg/24h (reference range, ≤6mg/24h). Urinary 5-HIAA has sensitivity of 73% and specificity of 100% for carcinoid tumors [8]. 5-HIAA is the metabolic product of serotonin. The patient’s serotonin and chromogranin A levels are given in Table 1. Chromogranin A has sensitivity of 62.9% and specificity of 98.4% for carcinoid tumors [9-11]. Serotonin is broken down by the liver and excreted in urine as 5-HIAA. A metastatic, poorly differentiated, stage IV carcinoid tumor was diagnosed on the basis of liver and bone metastases and a right lower lobe lung nodule with no defined primary site. In order to identify the primary site of the carcinoid tumor, whole-body octreoscan imaging with planar single-photon emission computed tomography was performed, which showed no evidence of octreotide-avid malignancy. The liver lesions visualized on the recent PET-CT scan were “cold” on the octreotide scan.

**Table 1:** Serotonin and chromogranin A levels in our patient

	**July 2012 (at diagnosis)**	**October 2012 (after chemotherapy)**	**November 2012 (after first HAE)**	**December 2012 (after second HAE)**	**April 2013 (after cryoablation of right shoulder)**	**Reference range (ng/ml)**
Serotonin	1,322	554	278	–	825	22 to 180
Chromogranin A	148	210	152	150	98	1.9 to 15

HAE, Hepatic artery embolization.

The goals of treating high-grade, poorly differentiated carcinoid tumors of unknown origin are decreasing the tumor load and palliation of symptoms. Our patient was offered chemotherapy with cisplatin and etoposide. At her follow-up examination after two cycles of chemotherapy, the patient had some improvement in pain and better range of motion of the right shoulder. A repeat PET scan taken after two cycles of chemotherapy showed a mixed response to chemotherapy. The standardized uptake value (SUV) of the right scapular lesion had diminished from 6.4 to 5.4. The SUV of the right side of the vertebral body at T8 had decreased from 4.8 to 4.4. Diffuse, generalized hepatic metastases had increased in size, and there was one new 1.5cm hypermetabolic right-lower-lobe pulmonary nodule. The patient finished four cycles of chemotherapy with cisplatin and etoposide. A CT scan of the abdomen after chemotherapy showed bulky, centrally necrotic liver metastases with nearly total involvement of the left hepatic lobe and pulmonary metastases within the right lung abutting the mediastinal pleura, indicating an inadequate response to treatment. The patient still had high serotonin and chromogranin A levels (Table 1). Therefore, hepatic artery embolization (HAE) of the right hepatic artery was performed, along with administration of octreotide to mitigate tumor burden from liver metastases. The patient’s disease activity decreased, as indicated by the biochemical markers listed in Table 1, although her chromogranin A level was still elevated.

One month later, the patient also received embolization of the left hepatic artery with a second dose of octreotide. Her hepatomegaly decreased. Symptomatically, she felt better. Her liver disease responded favorably to embolization. Post-embolization syndrome with nausea and low-grade fever resolved with symptomatic treatment with anti-nausea medication, hydration and a short-term hospital stay after she underwent a second HAE. At a follow-up examination after her second HAE, the patient complained of pain in her right shoulder, which was graded 5 of 10 in severity with weakness and reduced range of motion. Cryoablation of the right shoulder was offered to the patient. A CT scan of her right upper extremity showed asymmetric atrophy involving the musculature of the right rotator cuff, including the subscapularis, supraspinatus and infraspinatus muscles as well as the right deltoid muscle. Increased lytic destruction involving the inferior part of the right glenoid was observed, with a tumor extending through the posterior cortex at the spinoglenoid notch. The patient underwent cryoablation of the right shoulder, which resulted in partial relief of the pain. Three months after embolization of the left hepatic artery, CT scans of the abdomen with and without contrast were repeated, which showed metastases in the medial left hepatic lobe and the posterior right hepatic lobe. The patient underwent embolization of right hepatic artery, including the cystic artery. Tumor activity in this case was resistant to treatment, requiring multiple episodes of local modality treatments with HAE. The patient also developed a soft-tissue mass on the left posterior chest wall about 2cm×2cm in size. An excisional biopsy of the mass showed a metastatic, poorly differentiated neuroendocrine tumor. Unfortunately, despite systemic chemotherapy, repeated HAE and cryoablation of the right shoulder, the pain in the patient’s right shoulder remained to a certain extent. She had no systemic symptoms of carcinoid syndrome, such as diarrhea, flushing, cardiac arrhythmia and shortness of breath, throughout the disease course. She eventually died as a result of liver failure.

## Discussion

Carcinoid tumors are neuroendocrine tumors derived from enterochromaffin cells, also called Kulchitsky cells. Neuroendocrine cells are distributed throughout the whole body, have a similar histological appearance upon diagnosis and can release many neurosecretory granules, such as serotonin, gastrin, somatostatin and substance P. The capacity to secrete a variety of neuropeptides from enterochromaffin cells triggers the symptoms. Depending on the location of the primary carcinoid tumor with or without metastases, symptoms of carcinoid tumors can vary. Although a majority of carcinoid tumors have indolent disease activity, they can have an aggressive disease course different from classic carcinoid syndrome, as we observed in our patient. The 5-year overall survival rate for carcinoid tumor patients, regardless of site, is 67% [12]. If there are distant metastases to bone, liver and other organs, the 5-year survival rate ranges from 4% to 35% [13]. Our present case report describes a patient who was incidentally diagnosed with malignant carcinoid tumor with high-grade features and lung, bone and liver metastases of unknown origin.

Poorly differentiated neuroendocrine cancer is aggressive in nature and it can present with multiple metastases, including liver, bone, lung and brain, at the onset of presentation. It is usually locally aggressive and becomes unresectable. In such cases, investigation for the primary site of cancer origin may involve extensive imaging with contrast-enhanced CT or MRI scans, octreotide scans, capsule endoscopy, endoscopic ultrasound and even laparoscopic surgery. However, the decision to order several diagnostic tests for the detection of the primary site should be tailored specifically for each patient. Our patient had a negative octreotide scan, and we were unable to detect the primary site by using this specialized imaging technique. A negative octreotide scan implies that tumor cells may not have somatostatin receptors. In our patient, this explained why she still had a heavy tumor burden and did not respond adequately to octreotide.

At the time of her presentation, our patient did not have any symptoms other than right shoulder pain precipitated by a fall. She was found to have numerous liver metastases with poorly differentiated neuroendocrine features, indicating a poor prognosis, thus she was recommended to undergo systemic chemotherapy with cisplatin and etoposide. Even though treatment with systemic chemotherapy is neither curative nor effective, Fjällskog *et al.* reported that treatment with cisplatin and etoposide led to radiological and/or biochemical responses in 56% of 18 patients with foregut carcinoids and 50% of 14 patients with malignant endocrine pancreatic tumors, with a median response duration of 9 months [14]. Mitry *et al.* confirmed that poorly differentiated neuroendocrine tumors respond to the cytotoxic agents cisplatin and etoposide, although the 2-year survival is less than 20% [15]. Therefore, patients can be effectively palliated by cisplatin and etoposide treatment without significant morbidity. As shown in our present case, an interventional radiology–guided procedure partially relieved the patient’s symptoms of pain in the right shoulder. Until more effective chemotherapy is developed, palliation with chemotherapy is the goal.

Our patient had some improvement in her right shoulder pain and better range of motion after two cycles of chemotherapy. During chemotherapy, she experienced side effects of diarrhea and poor appetite. She also underwent repeated HAE for numerous liver metastases, which persisted despite receiving systemic chemotherapy. Our patient had no symptoms of carcinoid syndrome, despite the involvement of large liver metastases.

Metastatic carcinoid tumors can have either osteoblastic or osteolytic lesions, with the latter thought to carry a worse prognosis [7]. Our patient had permeative destruction of the right scapula at her initial presentation, which could have been one of the adverse prognostic factors. This illustrates the need for repeated HAE to reduce tumor burden in our patient. Our patient also developed skin metastasis to a left chest wall lesion. The manifestation of skin metastases from carcinoid tumors has been reported in the past and is usually a sign of advanced disease.

## Conclusion

Carcinoid tumors can arise from any epithelial cell throughout the whole body and they can exhibit neuroendocrine behavior, although they are typically recognized on the basis of gastrointestinal complaints. The case of our patient demonstrates a rare initial clinical presentation of metastatic carcinoid tumor with right shoulder pain after the patient had a fall. The diagnosis was challenging. The patient was treated with palliative chemotherapy, locoregional treatment with HAE and cryoablation of the shoulder. She had partial relief of her right shoulder pain and relief of cachexia, as well as improved quality of life with the above treatments, before she died less than 2 years after her diagnosis.

## Consent

Written informed consent for publication could not be obtained from our deceased patient's next of kin despite all reasonable attempts. Every effort has been made to protect the identity of our patient and ensure anonymity.

## Abbreviations

5-HIAA: 5-Hydroxyindoleacetic acid; HAE: Hepatic artery embolization.

## Competing interests

The authors declare that they have no competing interests.

## Authors’ contributions

NT wrote the manuscript. RO edited the manuscript and provided suggestions. Both authors read and approved the final manuscript.
